# In Vitro Capacitation in Boar Sperm: Evaluation of Selected Detection Techniques

**DOI:** 10.3390/mps9030098

**Published:** 2026-06-15

**Authors:** Barbora Klusackova, Zuzana Pilsova, Katerina Nemeckova, Aneta Pilsova, Pavla Postlerova

**Affiliations:** Department of Veterinary Sciences, Faculty of Agrobiology, Food, and Natural Resources, Czech University of Life Sciences Prague, 165 00 Prague, Czech Republic; klusackovab@af.czu.cz (B.K.); pilsovaz@af.czu.cz (Z.P.); nemeckovak@af.czu.cz (K.N.); pilsova@af.czu.cz (A.P.)

**Keywords:** phosphotyrosine, phosphorylation, acrosome reaction, acrosin, calcium

## Abstract

Sperm capacitation is essential for fertilization and involves coordinated changes in membrane organization, ion fluxes, and intracellular signaling. However, commonly used detection methods may reflect different biological events, which can be strongly influenced by experimental methodology. This study critically evaluated fluorescence-based approaches for assessing capacitation in boar spermatozoa, focusing on their specificity, interpretative limits, and methodological sensitivity. Ejaculated boar spermatozoa were incubated under in vitro capacitating conditions in TALP medium. Selected samples were subsequently treated with calcium ionophore to induce the acrosome reaction (AR). Phosphotyrosine (PTyr) immunofluorescence was assessed using five fixation and labeling protocols, acrosin redistribution was evaluated with the ACR.2 antibody, calcium ion redistribution was assessed using chlortetracycline (CTC) fluorescence, and acrosomal responsiveness was monitored by peanut agglutinin (PNA) lectin labeling. PTyr immunofluorescence was highly dependent on fixation protocol, indicating marked methodological sensitivity. Acrosin immunodetection revealed a clear capacitation-associated redistribution from weak or diffuse staining to a well-defined acrosomal pattern, whereas ionophore treatment caused a pronounced signal loss consistent with acrosomal exocytosis. PNA labeling confirmed that capacitation alone did not increase spontaneous acrosome loss, whereas ionophore treatment induced a robust AR. CTC staining showed a significant shift from whole-head pattern to acrosome in TALP-treated spermatozoa, indicating capacitation-associated Ca^2+^ redistribution. Together with CTC and Western blot data, these findings show that sperm capacitation status should be evaluated using multiple complementary markers rather than a single gold-standard assay.

## 1. Introduction

Sperm capacitation is a functional maturation process that enables mammalian spermatozoa to acquire fertilizing competence. In vivo, this process occurs within the female reproductive tract [1,2,3]. Under laboratory conditions, it is commonly induced by incubation in defined capacitation media [4,5]. Capacitation is represented by cholesterol efflux from the sperm plasma membrane, leading to increased membrane fluidity [6], as well as ion fluxes such as Ca^2+^ influx [7] and Zn^2+^ efflux [8]. Intracellular signaling pathways are activated, particularly the cyclic adenosine monophosphate/protein kinase A (cAMP–PKA) cascade, increased protein phosphorylation, including tyrosine phosphorylation [9]. These processes are accompanied by reorganization of sperm membrane domains [10], actin polymerization [11], and changes in mitochondrial activity [12] and motility patterns, including the development of hyperactivation [13]. Together, these events prime the spermatozoa for the acrosome reaction (AR) and enable successful interaction with the oocyte.

Various methods are employed to detect these changes in boar spermatozoa, each focusing on different functional aspects with varying reliability [14]. A critical change during capacitation is the efflux of Zn^2+^, which is essential for Ca^2+^ influx into the sperm cell. Zn^2+^ blocks the voltage-gated hydrogen channel 1 HVCN1, which, when activated, allows Ca^2+^ to enter through the cation channel of sperm (CatSper), crucial for capacitation and, along with that, hyperactivation of motility [8,15]. The intracellular calcium concentration [Ca^2+^]_i_ increases during capacitation. It can be detected using chlortetracycline (CTC) staining, a fluorescent dye that forms chelate complexes of Ca^2+^. This method enables precise determination of the time at which the largest number of spermatozoa are in a capacitated state using immunofluorescence and flow cytometry [14,16,17].

There are several pathways leading to tyrosine phosphorylation (PTyr) of several sperm proteins [18]. PTyr increases during capacitation and can be detected by Western blotting and indirect immunofluorescence using specific antibodies. These methods reveal changes in the distribution and intensity of phosphorylated proteins, providing insights into the stages of capacitation [2,19,20,21,22]. However, the PTyr is widely influenced by the methodological approach, such as the fixation protocols, or strongly influenced by them [23], as well as by the employed capacitation medium (CM) [5].

Capacitation also leads to the activation and release of acrosomal enzymes, such as acrosin, which are crucial for binding to and penetrating the zona pellucida. Acrosin is present in ejaculated spermatozoa as the inactive precursor which converts to proacrosin during capacitation, which, in turn, converts to acrosin during capacitation. A specific primary antibody against acrosin can detect this change, with a stronger antibody signal in capacitated spermatozoa [14,24]. Visualizing the acrosome with PNA lectin conjugated with a fluorescent probe, which binds to glycans on the outer acrosomal membrane, allows for differentiation between acrosome-intact and acrosome-reacted sperm. In vitro induction of the AR using calcium ionophore (CaI) provides insight into the proportion of spermatozoa functionally primed for acrosomal exocytosis, and thus indirectly reflects the fraction of cells that have undergone capacitation [25,26].

Although these detection methods are highly specialized and robust, each of them reflects only a single aspect of the complex capacitation process. Consequently, individual assays are not sufficient on their own to clearly distinguish capacitated from non-capacitated spermatozoa, since the detected parameters may be affected by other variables, including the composition of the capacitation media and the methodological steps preceding the analysis [23]. Therefore, the aim of this study was to evaluate selected complementary approaches for assessing sperm capacitation status in a widely used CM, with particular emphasis on the impact of fixation conditions on PTyr detection and on the interpretation of capacitation-associated changes. Specifically, we compared CTC staining, PTyr detection under different fixation protocols, Western blot analysis of PTyr patterns, and assessment of the AR using PNA labeling to define their reliability and methodological limitations under controlled experimental conditions.

## 2. Materials and Methods

All chemicals used in this study were obtained from Sigma-Aldrich (St. Louis, MO, USA), unless otherwise specified. Ejaculates from 29 fertile Duroc boars aged 12–18 months were sourced from the Insemination Station Skrsin (LIPRA PORK a.s., Rovensko pod Troskami, Czech Republic), where only boars with proven fertility are routinely used for semen collection. Spermatozoa were transported to the laboratory in a styrofoam box maintained at 37 °C and processed within 3 h of collection. Before experimental processing, sperm concentration and motility were evaluated using standard andrological techniques with a light microscope. Only ejaculates showing normal microscopic appearance and at least 70% total motility were included in the study. All received ejaculates fulfilled these criteria. The initial sperm concentration of the received ejaculates ranged from 20 × 10^7^ to 100 × 10^7^ sperm/mL.

A total of 65 ejaculate samples from 29 boars were included in the study. Although some boars were sampled repeatedly, all samples were obtained from separate ejaculate collections. For each biological replicate, ejaculates from three fertile adult boars were diluted in PBS (phosphate-buffered saline, #P4417) to a concentration of 5 × 10^7^ sperm/mL and pooled to eliminate individual variations. The remaining seminal plasma was removed by washing the spermatozoa three times in PBS and centrifugation at 300× *g* for 5 min. Non-capacitated spermatozoa (Non-cap; 5 × 10^7^) were stored in pellets at −20 °C for gel electrophoresis.

### 2.1. Sperm In Vitro Capacitation (IVC) and Acrosome Reaction (AR)

Spermatozoa were then diluted in TALP CM (pH 7.4) [114 mM NaCl (#SZBC1650V), 3.2 mM KCl (#P-5405), 0.35 mM NaH_2_PO_4_ (#S9638), 18 mM Na-Lactate (#L4263), 8 mM Ca-Lactate (#C8356), 0.5 mM MgCl_2_ (#M2393), 1.1 mM Na-pyruvate (#P2256), 0.17 mM Kanamycin sulfate (#606-15), 1% (*w*/*v*) polyvinyl alcohol (PVA; #P8136), 5 mM Glucose (#G-7021), 3% (*w*/*v*) bovine serum albumin (BSA; #A9647), 25 mM NaHCO_3_ (#S5761), 2 mM Caffeine (#C0750)] [27]. Spermatozoa underwent capacitation for 3 h at 37 °C with 5% (*v*/*v*) CO_2_. Capacitated spermatozoa (Cap; 5 × 10^7^) were stored in pellets at −20 °C for gel electrophoresis. Part of the IVC sperm suspension (5 × 10^7^ sperm/mL) was supplemented with calcium ionophore (CaI) A23187 (#C7522) to a final concentration of 5 µM (Cap + CaI). To assess whether CaI alone could induce acrosomal exocytosis in the absence of prior IVC, an aliquot of the non-capacitated sperm suspension (5 × 10^7^ sperm/mL) in non-capacitation medium (TALP NCM; pH 7.4; 114 mM NaCl, 3.2 mM KCl, 0.35 mM NaH_2_PO_4_, 18 mM Na-Lactate, 8 mM Ca-Lactate, 0.5 mM MgCl_2_, 0.17 mM Kanamycin sulfate, 1% (*w*/*v*) PVA, 5 mM Glucose) was supplemented with A23187 (#C7522) to a final concentration of 5 µM (Non-cap + CaI). CaI was prepared as a 5 mM stock solution in ethanol, resulting in a final ethanol concentration of 0.1% (*v*/*v*) in the sperm suspension. These samples (Cap + CaI and Non-cap + CaI) were then incubated for 1 h at 37 °C in 5% CO_2_ to induce the AR in vitro. Vehicle controls with 0.1% (*v*/*v*) ethanol concentration were employed (Non-cap + EtOH, Cap + EtOH).

### 2.2. Detection of PTyr-Specific Immunofluorescence Patterns with the Use of Distinct Fixation Protocols

Non-capacitated (Non-cap) and IVC (Cap) spermatozoa were divided into five groups: (i): spermatozoa labeled with primary antibody in suspension and then fixed with 70% acetone/methanol (1:1, *v*/*v*) (Susp.), (ii) fixed in suspension in 2% formaldehyde methanol-free (Carl Roth GmbH, Karlsruhe, Germany, Art.-Nr. 4235.1) with 2% BSA and labeled with antibody on slides (F mf), (iii): fixed in suspension in 2% formaldehyde with 2% BSA and labeled with antibody on slides (F), (iv): fixed in 70% acetone and labeled with antibody on slides (A), (v): fixed in 70% methanol and labeled with antibody on slides (M).

Spermatozoa (Non-cap and Cap) of the first group (i) were labeled with a mouse anti-phosphotyrosine antibody (clone 4G10; Millipore, MA, USA) diluted 1:150 for 1 h in suspension. Non-cap spermatozoa were incubated with the antibody in PBS supplemented with 2% BSA at room temperature (RT). After 2 h of IVC, the 4G10 antibody was added to the sperm suspension (final dilution 1:150) in TALP CM, and the spermatozoa were further incubated for 1 h at 37 °C with 5% (*v*/*v*) CO_2_. After incubation, spermatozoa were washed 3 times with PBS (300× *g* for 5 min) and resuspended in PBS. Sperm suspension was placed onto slides and fixed with 70% acetone/methanol.

In other experimental groups (ii–v), Non-cap and Cap sperm suspensions were centrifuged at 300× *g* for 10 min. The supernatant was discarded, and the spermatozoa were fixed. Spermatozoa fixed in suspension (ii, iii) were resuspended in 2% formaldehyde with 2% BSA and in 2% formaldehyde methanol-free (#4235.1, Carl Roth GmbH, Karlsruhe, Germany) with 2% BSA, respectively. Spermatozoa were incubated for 20 min at RT. Then, the samples were washed three times with PBS (300× *g* for 5 min) and resuspended in PBS. Sperm suspensions were placed onto slides and allowed to dry. Sperm suspensions fixed onto the slides (iv, v) were placed on slides and fixed with 70% acetone and 70% methanol, respectively.

Slides were then rinsed with PBS and incubated with SuperBlock Blocking Buffer (#37517, ThermoFisher Scientific, Rockford, IL, USA) for 30 min. Four groups of slides (ii–v) were then treated with 4G10 antibody diluted 1:300 in PBS at 4 °C in a wet chamber overnight. After washing with PBS, all slides were labeled with secondary antibody Goat anti-Mouse IgG (H + L) Highly Cross-Adsorbed conjugated to Alexa Fluor^®^ Plus 488 (#A11001, Invitrogen, Rockford, IL, USA) diluted 1:300 in PBS and incubated at 4 °C for 1 h in the dark in a wet chamber. Slides were then washed with PBS and treated with PNA conjugated with rhodamine (#RL-1072-5, Vector Laboratories, Newark, CA, USA), diluted 1:1000 in PBS at RT, in a wet chamber in the dark for 30 min. After washing with PBS and water, sperm samples were mounted in Vectashield mounting medium containing DAPI (#H-1200, Vector Laboratories, Newark, CA, USA).

Sperm fluorescence was analyzed under a confocal microscope (Zeiss LSM 800; Carl Zeiss AG, Oberkochen, Germany) using NIS-Elements AR 4.30.01 software (Nikon Instruments Inc., NY, USA). At least 100 spermatozoa per sample were subjectively evaluated, and all the detected patterns were counted. For image analysis, only spermatozoa with intact acrosomes were included.

### 2.3. SDS Gel Electrophoresis and Protein Blotting

Frozen sperm pellets from Non-cap and Cap groups were lysed in 100 µL of 2× reducing Laemmli buffer containing 20% (*v*/*v*) glycerol (#G9012), 4% (*w*/*v*) SDS (sodium dodecyl sulfate; #161-0732), 0.125 M Tris-HCl (pH 6.8; #161-0799), 5% (*v*/*v*) β-mercaptoethanol (#M7522), and 0.005% (*w*/*v*) bromophenol blue (#B0126), supplemented with protease and phosphatase inhibitors (#11873580001, Roche, Mannheim, Germany). Samples were incubated on ice for 30 min with intermittent mixing and then boiled for 5 min. Proteins were separated by SDS-PAGE on 12% polyacrylamide gels (12% (*w*/*v*) Acrylamide/Bis-acrylamide solution (Bio-Rad, Hercules, CA, USA); 1.5M Tris-HCl (Bio-Rad, Hercules, CA, USA), pH 8.8; 0.1% (*w*/*v*) SDS; TEMED; 0.1% (*w*/*v*) ammonium persulfate) and 4% stacking gel (4% Acrylamide/Bis-Acrylamide solution; 0.5M Tris-HCl pH 6.8 (Bio-Rad, Hercules, CA, USA); 0.1% SDS; TEMED; 0.1% ammonium persulfate) using Precision Plus Protein All Blue Standards (#1610373, Bio-Rad, Hercules, CA, USA) and transferred onto nitrocellulose membranes (#GE10600001, Amersham Protran, GE Healthcare, Chicago, IL, USA) at 0.5 A for 1 h. Membranes were blocked in 5% (*w*/*v*) non-fat dry milk (Blotto; #SC-2325, ChemCruz, Santa Cruz Biotechnology, Inc., Dallas, TX, USA) prepared in PBS and incubated overnight at 4 °C with anti-phosphotyrosine antibody (clone 4G10; 1:1000) or mouse IgG isotype control (#31903, ThermoFisher Scientific, Waltham, MA, USA; 1:10,000). For densitometric analysis, the membranes were incubated with mouse anti-tubulin antibody (clone DM1A; Invitrogen, Rockford, IL, USA) diluted 1:1000 in PBS. After washing in PBS-T (PBS containing 0.1% [*v*/*v*] Tween-20), membranes were incubated with horseradish peroxidase-conjugated goat anti-mouse IgG secondary antibody (Bio-Rad; 1:3000) for 1 h at room temperature. Immunoreactive signals were detected using SuperSignal West Pico PLUS substrate (#34580, Thermo Fisher Scientific, Rockford, IL, USA), visualized with an Azure C300 imaging system (Azure Biosystems, Dublin, CA, USA), and quantified using IMAGE Studio Digits 3.1 software (Li-COR Biotechnology, Lincoln, NE, USA).

### 2.4. Detection of Acrosin-Specific Immunofluorescence Patterns

Non-cap and Cap sperm suspensions were centrifuged at 300× *g* for 10 min. The supernatant was discarded, spermatozoa were further washed twice in PBS with centrifugation at 300× *g* for 10 min, and then resuspended in PBS. Sperm suspension was placed onto slides and fixed with 70% acetone. Slides were rinsed with PBS and incubated with SuperBlock Blocking Buffer for 30 min. Slides were then treated with mouse ACR.2 (#11-260-C100, Exbio, Prague, Czech Republic) diluted 1:100 in PBS at 4 °C in a wet chamber for 1 h. After washing with PBS, all slides were labeled with secondary antibody Goat anti-Mouse IgG (H + L) Highly Cross-Adsorbed conjugated to Alexa Fluor^®^ Plus 488 (Invitrogen, CA, USA), diluted 1:300 in PBS and incubated at 4 °C for 1 h in the dark in a wet chamber. Slides were then washed with PBS and treated with PNA conjugated with rhodamine (Vector Laboratories, Newark, CA, USA) diluted 1:500 in PBS at RT in a wet chamber in the dark for 30 min. After washing with PBS and water, sperm samples were mounted in Vectashield mounting medium containing DAPI (Vector Laboratories, Newark, CA, USA).

Sperm fluorescence was analyzed under a Nikon Eclipse Ni-U microscope (Nikon, Tokyo, Japan) using constant settings in NIS-Elements software. At least 100 spermatozoa per sample were subjectively evaluated and classified as Non-cap (low fluorescent signal) or Cap (fluorescence restricted to the acrosome) and AR (no fluorescent signal) according to Ded et al. (2019) [14].

### 2.5. Detection of Capacitation-Associated Calcium Distribution Changes

Sperm capacitation was assessed using the chlortetracycline (CTC) fluorescence assay. The CTC staining solution consisted of 0.78 mM chlortetracycline (#C-4881) dissolved in 20 mM Tris, 130 mM NaCl, and 5 mM L-cysteine (pH 7.8). Briefly, 30 µL of sperm suspension from the Non-cap and Cap sperm groups (5 × 10^7^ spermatozoa/mL) were mixed with 30 µL of CTC solution and incubated for 10 min at room temperature in the dark. The reaction was then stopped by adding 20 µL of 4% paraformaldehyde. Subsequently, 10 µL of the suspension was mounted in VectaShield containing 4′,6-diamidino-2-phenylindole (DAPI; #H-1200, Vector Laboratories, Newark, CA, USA). Sperm fluorescence was analyzed under a confocal microscope (Zeiss LSM 800; Carl Zeiss AG, Oberkochen, Germany) using NIS-Elements AR 4.30.01 software (Nikon Instruments Inc.). At least 100 spermatozoa per sample were assessed by subjective scoring and classified either as non-capacitated, characterized by uniform fluorescence over the entire sperm head, or as undergoing capacitation-associated changes, characterized by fluorescence restricted to the acrosomal region, according to Ded et al. (2019) [14].

### 2.6. Detection of Acrosome Reaction

Non-cap, Non-cap + EtOH, Non-cap + CaI, Cap, Cap + EtOH, and Cap + CaI sperm suspensions were centrifuged at 300× *g* for 5 min. The supernatant was discarded, and spermatozoa were further washed twice in PBS with centrifugation at 300× *g* for 5 min. Spermatozoa were resuspended in PBS. Sperm suspension was placed onto slides and fixed with 70% acetone. Slides were then washed with PBS and treated with PNA conjugated with rhodamine (Vector Laboratories) diluted 1:500 in PBS at RT in a wet chamber in the dark for 30 min. After washing with PBS and water, sperm samples were mounted with VectaShield mounting medium with DAPI (Vector Laboratories).

Sperm fluorescence was analyzed under a confocal microscope (Zeiss LSM 800; Carl Zeiss AG) using NIS-Elements AR 4.30.01 software (Nikon Instruments Inc.). At least 100 spermatozoa per sample were subjectively evaluated, and all the detected patterns were counted.

### 2.7. Data and Statistical Analysis

When counting spermatozoa from a confocal or epifluorescence microscope, at least 100 were counted in each sample. For PTyr and acrosin detection, spermatozoa with PNA labeling in the acrosome were counted for Non-cap and Cap spermatozoa. In the case of acrosin detection after AR, only spermatozoa without a PNA signal in the acrosome were counted. When detecting AR using PNA labeling, all spermatozoa in the samples were counted, regardless of their acrosome status.

For statistical analyses, GraphPad Prism version 9.0 (GraphPad Software, Boston, MA, USA) was used. Data normality was assessed using the Shapiro–Wilk test prior to parametric analysis. The effects of IVC and in vitro AR, and, for PTyr analysis, the effect of fixation treatment, as well as their interaction, were evaluated using two-way analysis of variance (two-way ANOVA). When significant main effects or interactions were detected, post hoc multiple comparisons were performed using Tukey’s multiple comparison test to identify differences between experimental groups. The results are presented as mean ± standard error of the mean (SEM). Differences were considered statistically significant at *p* < 0.05.

## 3. Results

### 3.1. Fixatives Influence the Presentation of Specific PTyr Fluorescence Patterns

**Figure 1:**
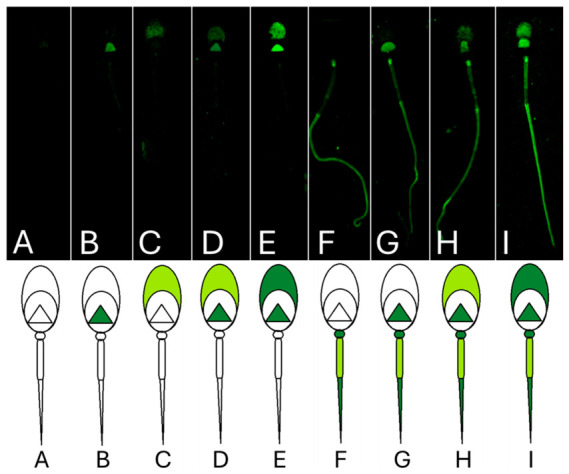
Schematic representation of detected fluorescence patterns when labeling with the 4G10 antibody. (A) no fluorescent signal, (B) strong fluorescent signal in the equatorial subsegment (EqSS), (C) weak fluorescent signal in the acrosome, (D) weak fluorescent signal in the acrosome and strong signal in EqSS, (E) strong fluorescent signal in the acrosome and EqSS, (F) strong fluorescent signal in the neck and main part of the flagellum and weaker signal in the midpiece, (G) strong fluorescent signal in the EqSS, neck, and main part of the flagellum and weaker signal in the midpiece, (H) strong fluorescent signal in EqSS, weaker signal in the acrosome, strong signal in the neck and main part of the flagellum, and weaker signal in the midpiece, (I) strong fluorescent signal in EqSS, acrosome, neck, and main part of the flagellum, and weaker signal in the midpiece.

Indirect immunofluorescence using sperm labeling with an antibody against phosphotyrosine is often used as a typical marker of capacitation. We compared the effect of different fixatives on the presentation of specific fluorescence patterns. Two aldehyde fixatives (2% formaldehyde—F—and 2% methanol-free formaldehyde—F mf) and two alcohol fixatives (70% acetone—A—and 70% methanol—M) were chosen. In addition, we evaluated spermatozoa labeled with the antibody in suspension, which we subsequently fixed with acetone/methanol (Susp.). In total, nine different fluorescence patterns (Figure 1) were detected.

**Figure 2:**
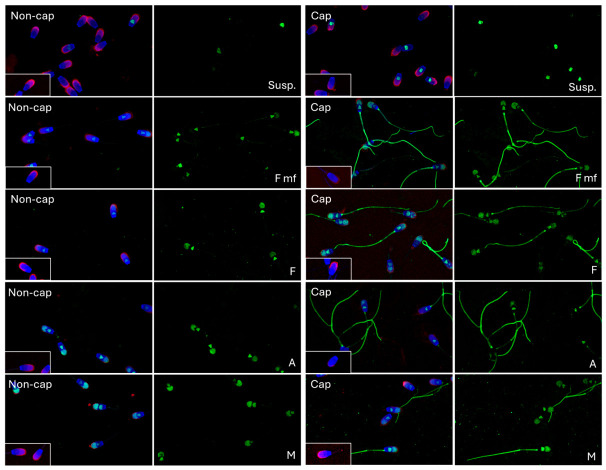
Phosphotyrosine fluorescent patterns in boar spermatozoa fixed in different fixatives. Representative images of indirect immunofluorescence of non-capacitated (Non-cap) and capacitated (Cap) spermatozoa; Susp.—spermatozoa labeled with 4G10 antibody in suspension, F mf—spermatozoa fixed in 2% formaldehyde methanol free with 2% BSA in water, F—spermatozoa fixed in 2% formaldehyde with 2% BSA in water, A—spermatozoa fixed with 70% acetone, M—spermatozoa fixed with 70% methanol; green color (AlexaFluor 488)—immunofluorescence presenting the reaction of the antibody with antigen, red color (Rhodamine)—PNA-lectin staining of sperm acrosome, blue color (DAPI)—staining of the cell nucleus; negative controls are inserted in corners.

**Figure 3:**
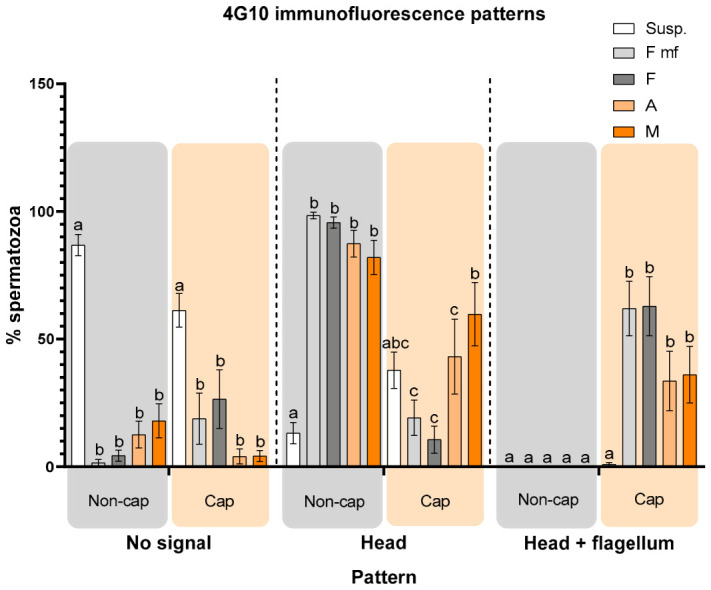
Graph showing the percentage of spermatozoa presenting different immunofluorescent patterns (No signal—no fluorescent signal, Head—fluorescent signal present in various compartments of sperm head, Head + flagellum—fluorescent signal present in various compartments of sperm head and in the flagellum); the graph compares differences between non-capacitated (Non-cap) and capacitated (Cap) spermatozoa within individual fixative (Susp., F mf, F, A, M). Error bars show SEM; statistically significant differences (*p* < 0.05) are indicated by letters. Dashed vertical lines separate the individual immunofluorescence patterns. Statistical comparisons were performed only within each pattern category. *n* = 5 independent biological replicates, each replicate consisted of a pooled ejaculate from three boars.

We further merged these patterns into three groups (No signal—no fluorescent signal, Head—fluorescent signal present in various compartments of the sperm head, Head + flagellum—fluorescent signal present in various compartments of the sperm head and in the flagellum). After defining these overarching groups, we detected significant differences between Non-cap and Cap spermatozoa across three fixation methods (2% formaldehyde, 2% formaldehyde methanol-free, and acetone). We did not detect any significant differences in the suspension (Susp.) group; both the Non-cap and Cap sperm groups showed no fluorescent signal in most cases (87% and 61%, respectively). We also did not find any significant differences in the methanol (M) fixed group; spermatozoa in this experimental group largely showed the Head pattern (Non-cap group in 82% and Cap group in 60%). Non-cap spermatozoa from the 2% formaldehyde (F), 2% formaldehyde methanol-free (F mf), and acetone (A) groups mainly showed a Head signal (95%, 98%, and 87%, respectively). In comparison, Cap spermatozoa showed a Head + flagellum signal (2% formaldehyde in 63%, 2% formaldehyde methanol-free in 62%, and acetone in 34%). The Head + flagellum pattern was not detected in any group of differently fixed Non-cap spermatozoa; so, it appears that this pattern is specific only to Cap spermatozoa. Figure 2 shows representative images of individual sperm groups, and a graph (Figure 3) shows the percentage representation of individual patterns.

**Figure 4:**
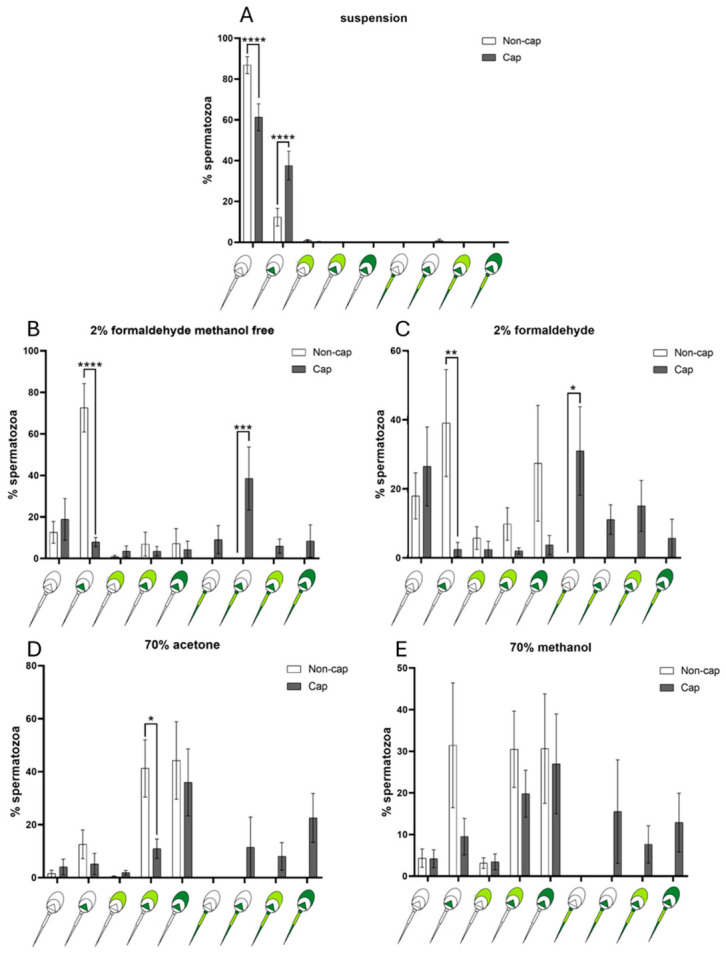
Phosphotyrosine fluorescent patterns in boar spermatozoa fixed with different fixatives. The graphs show the percentage of spermatozoa presenting different specific immunofluorescent patterns, as shown by the pictograms, and compare differences between non-capacitated (Non-cap) and capacitated (Cap) spermatozoa. (**A**) shows patterns after labeling the spermatozoa in suspension, (**B**) shows patterns in boar spermatozoa fixed in 2% formaldehyde methanol free with 2% BSA in water, (**C**) shows patterns in boar spermatozoa fixed in 2% formaldehyde with 2% BSA in water, (**D**) shows patterns in boar spermatozoa fixed in 70% acetone, and (**E**) shows patterns in boar spermatozoa fixed in 70% methanol. Error bars show SEM; statistically significant differences (*p* < 0.05) are indicated by an asterisks (* *p* < 0.05, ** *p* < 0.01, and **** *p* < 0.0001); *n* = 5 independent biological replicates, each replicate consisted of a pooled ejaculate from three boars.

Not all 9 detected patterns were found in all experimental sperm groups. The detailed percentage distribution of individual patterns across spermatozoa fixed using different fixatives is illustrated in Figure 4A–E. Panel A shows the representation of patterns in spermatozoa from the suspension PTyr antibody-labeled group. In this case, significant differences were detected in two expressed patterns. Non-cap spermatozoa showed a higher representation of the No-signal pattern (87%), and Cap spermatozoa showed significantly higher percentage of spermatozoa with strong fluorescence in EqSS (38%). Other PTyr sperm localizations were not detected using this antibody-labeling approach.

Panel B shows the representation of patterns in spermatozoa from the 2% formaldehyde methanol-free fixed group. Using this fixation, Non-cap spermatozoa typically showed a PTyr pattern with strong fluorescence in EqSS (73%), and Cap spermatozoa showed a pattern with strong fluorescence in EqSS, but in combination with fluorescence in the flagellum (39%). In both these patterns, a significant difference was observed between sperm groups at different maturation states.

Using 2% formaldehyde fixation (Panel C), variations in pattern assessment among individual samples were relatively extensive. Still, significant differences were detected only in the pattern with strong fluorescence in EqSS alone, which was typical for Non-cap spermatozoa (39%), and in the pattern with fluorescence exclusively in the flagellum, which was typical for Cap spermatozoa (31%).

Panel D shows the pattern representation when spermatozoa were fixed with 70% acetone. In this case, a significant difference was detected only in the pattern with weak fluorescence in the acrosome and strong fluorescence in the EqSS, which was more prevalent with significance (*p* < 0.05) in Non-cap spermatozoa (41%) than in Cap spermatozoa (11%). However, the pattern with strong fluorescence in the acrosome and EqSS was highly prevalent in both Non-cap spermatozoa (44%) and Cap spermatozoa (36%).

Spermatozoa fixed with 70% methanol (Panel E) showed no significant differences between Non-cap and Cap spermatozoa in any pattern. Although fluorescence patterns with signal in the flagellum were observed only in Cap spermatozoa, these groups showed substantial variation in pattern assessment across individual samples. Similarly, a decrease in the proportion of spermatozoa showing PTyr labeling exclusively in the EqSS region was observed after capacitation.

In summary, aldehyde fixatives reliably distinguish between non-capacitated and capacitated spermatozoa, with capacitated spermatozoa typically exhibiting a fluorescent signal in the flagellum. The same result can be achieved using 70% acetone. However, individual fixatives differ from one another, and the chosen fixative should always be considered when interpreting PTyr fluorescence as a marker of capacitation.

### 3.2. Increase in PTyr During IVC Detected by Electrophoresis and Western Blotting

**Figure 5:**
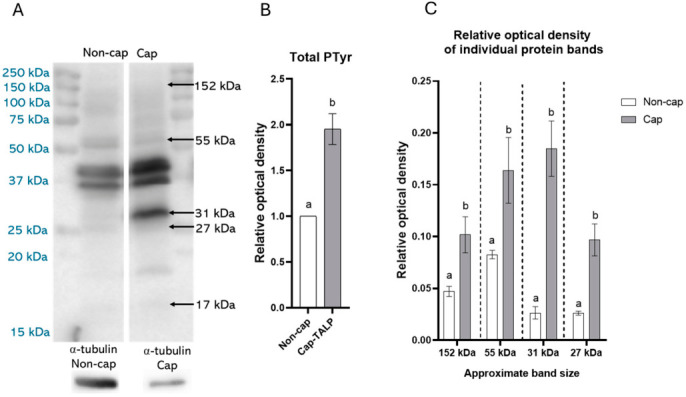
Phosphotyrosine (PTyr) detection in boar sperm protein extracts from non-capacitated (Non-cap) and capacitated (Cap) spermatozoa. Representative Western blot shows the molecular weights of detected phosphoprotein bands; the protein leader sizes are labeled in blue (**A**). Graphs show the relative optical density of Total PTyr (**B**) and individual protein bands (**C**). Protein loading was normalized to α-tubulin in sperm lysates. Error bars show SEM; statistically significant differences (*p* < 0.05) in total PTyr (**B**), or within one protein band (**C**), are indicated by letters. Dashed vertical lines in panel (**C**) separate the individual protein band evaluation. Statistical comparisons were performed only within each band size, comparing the Non-cap and Cap groups for the corresponding band. *n* = 6 independent biological replicates, each replicate consisted of a pooled ejaculate from three boars.

Differences in overall PTyr levels were detected between Non-cap and Cap spermatozoa (Figure 5). We observed a significant increase (*p* < 0.05) in total PTyr after IVC. We also detected four distinct PTyr-reactive bands with approximate molecular weights 152 kDa, 55 kDa, 31 kDa and 27 kDa with significant differences in relative optical density between Non-cap and Cap spermatozoa, with Cap spermatozoa showing in all of those protein bands significantly higher relative optical density. The largest difference was observed in the 31-kDa protein band.

### 3.3. Acrosin-Specific Immunofluorescence Patterns Differ Between Sperm Status

**Figure 6:**
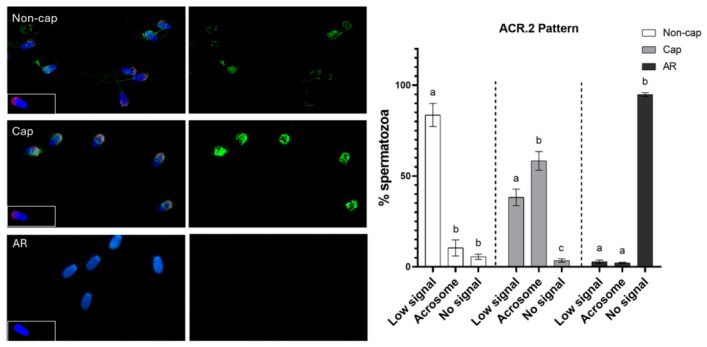
Immunofluorescent detection of acrosin in boar spermatozoa using ACR.2 antibody. Representative images of indirect immunofluorescence of non-capacitated (Non-cap), capacitated (Cap), and acrosome-reacted (AR) spermatozoa labeled with anti-ACR.2 antibody; green color (AlexaFluor 488)—immunofluorescence presenting the reaction of the antibody with antigen, red color (Rhodamine)—PNA-lectin staining of sperm acrosome, blue color (DAPI)—staining of the cell nucleus; negative controls are inserted in corners; distinct fluorescence patterns were classified as Low signal (weak or diffuse fluorescence), Acrosome (clear acrosomal localization), and No signal (absence of detectable fluorescence). The graph shows the percentage distribution of spermatozoa presenting individual ACR.2 fluorescence patterns in each experimental group (Non-cap, Cap, AR). Dashed vertical lines separate the evaluated categories. Error bars represent SEM; statistically significant differences between patterns within each experimental category (*p* < 0.05) are indicated by letters; *n* = 5 independent biological replicates, each replicate consisted of a pooled ejaculate from three boars.

Acrosin detection was evaluated by indirect immunofluorescence using the ACR.2 antibody in Non-cap, Cap, and AR spermatozoa (Figure 6). Based on signal detection, three distinct fluorescence patterns were identified: Low signal (weak or diffuse fluorescent signal), Acrosome (clear acrosomal fluorescent signal), and no signal (absence of detectable fluorescent signal).

In the Non-cap group, the predominant pattern was Low signal, observed in approximately 84% of spermatozoa. In contrast, the Acrosome pattern was detected in a minor proportion (around 10%), and no signal was rare (6%). Following capacitation, a significant shift in fluorescence distribution was observed. The proportion of spermatozoa exhibiting the Acrosome pattern increased markedly (approximately 58%), accompanied by a significant decrease in the Low signal pattern (38%). The No signal category remained minimal in this group. In contrast, acrosome-reacted (AR) spermatozoa showed a striking predominance of the No signal pattern (approximately 95%), consistent with the loss of acrosomal content following acrosomal exocytosis. Both the Low signal (3%) and Acrosome (2%) patterns were significantly reduced in AR spermatozoa compared to the Non-cap and Cap groups.

Statistical analysis confirmed significant differences in the distribution of fluorescence patterns among groups (*p* < 0.05). These findings indicate that ACR.2 immunolabeling reliably distinguishes between acrosome-intact and AR spermatozoa and reflects capacitation-associated redistribution of acrosin prior to acrosomal exocytosis.

### 3.4. Calcium Redistribution Is Evident After IVC

**Figure 7:**
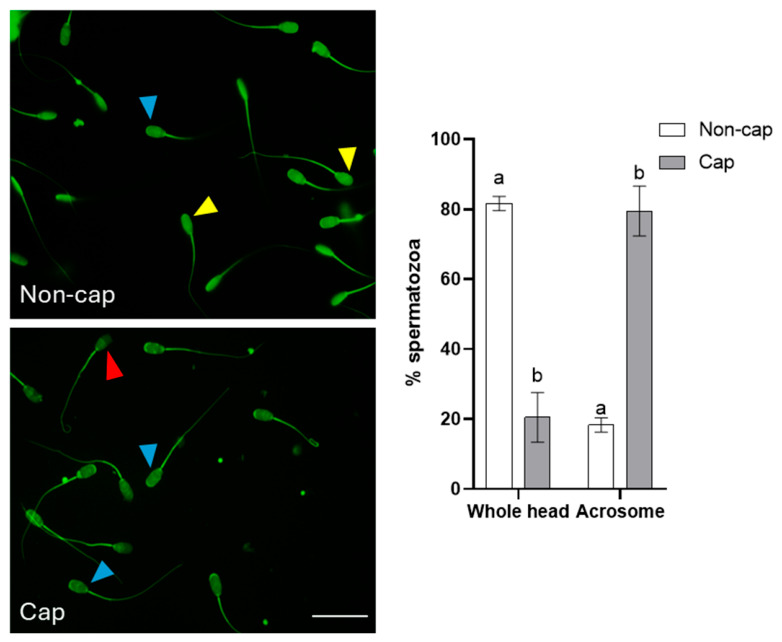
Detection of capacitation-associated changes in calcium distribution in Non-cap and Cap sperm samples using the chlortetracycline (CTC) assay. Yellow arrows indicate spermatozoa with strong fluorescence over the entire head (Whole head), blue arrows indicate spermatozoa with strong fluorescence over the acrosomal region and weaker fluorescence in the postacrosomal region (Acrosome), and red arrows indicate acrosome-reacted spermatozoa, which were excluded from the analysis. Scale bar = 20 µm. The graph shows the percentage of spermatozoa exhibiting specific fluorescence patterns, as illustrated in the representative images. Different letters (a, b) indicate statistically significant differences between individual patterns within each sperm group (*p* < 0.05); error bars represent SEM; *n* = 5 independent biological replicates, each replicate consisted of a pooled ejaculate from three boars.

Figure 7 presents the results of the fluorescent CTC assay, showing the proportion of spermatozoa exhibiting two predominant staining patterns: Whole head, characterized by strong fluorescence over the entire sperm head, and Acrosome, characterized by intense fluorescence in the acrosomal region with lower signal in the postacrosomal region. According to established interpretations of CTC staining [14], the Whole head pattern is typical of non-capacitated spermatozoa. In contrast, the Acrosome pattern reflects the capacitation-associated redistribution of intracellular Ca^2+^ and was therefore considered indicative of spermatozoa undergoing capacitation. The Whole head pattern predominated in the Non-cap group (81.7%), whereas it was significantly less frequent in the Cap-TALP group (20.5%; *p* < 0.0001). In contrast, the Acrosome pattern was detected in 18.3% of Non-cap spermatozoa and was markedly increased in the Cap-TALP group (79.5%). Together, these results confirm that incubation in Cap-TALP medium induced capacitation-associated calcium redistribution in boar spermatozoa.

### 3.5. Detection of Acrosome Reaction as an Indirect Marker of Capacitation

**Figure 8:**
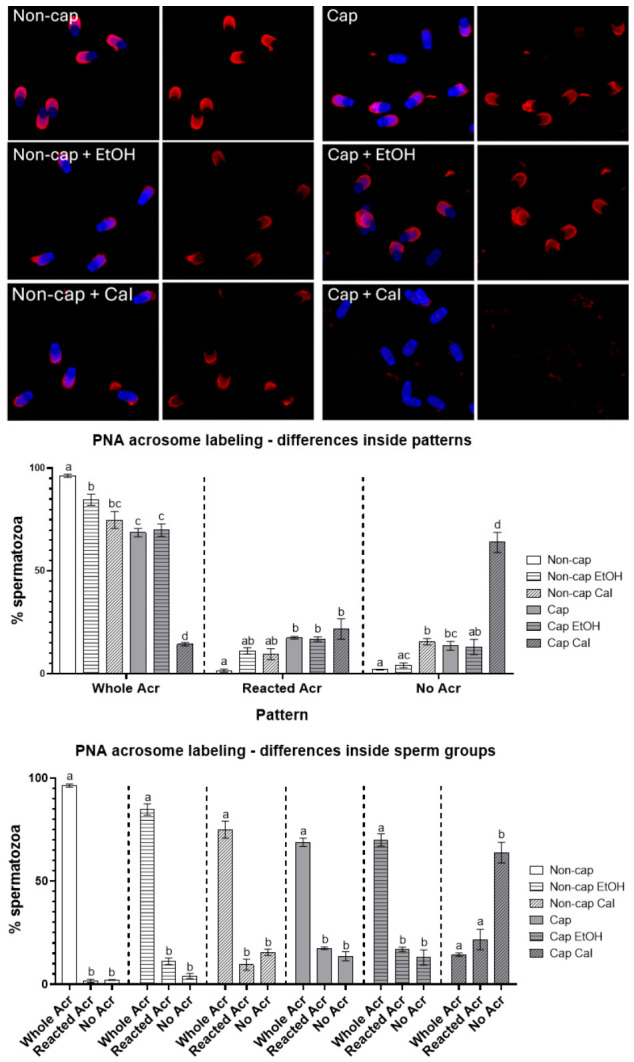
Fluorescent patterns of boar spermatozoa labeled with PNA lectin. Representative images of indirect immunofluorescence of non-capacitated spermatozoa (Non-cap), capacitated spermatozoa (Cap), non-capacitated spermatozoa incubated with 5 µM calcium ionophore (Non-cap + CaI), and capacitated spermatozoa incubated with 5 µM calcium ionophore (Cap + CaI); vehicle controls of 0.1% (*v*/*v*) ethanol are shown as Non-cap + EtOH and Cap + EtOH; red color (Rhodamine)—PNA-lectin staining of sperm acrosome, blue color (DAPI)—staining of the cell nucleus. Graphs show the percentage of spermatozoa presenting different immunofluorescent patterns (Whole Acr—clear fluorescent signal over the acrosome, Reacted—fluorescent signal showing ruptured acrosome, No Acr—no fluorescent signal present); the upper graph shows differences inside individual fluorescent patterns, the graph bellow shows differences inside each sperm group; error bars show SEM; statistically significant differences (*p* < 0.05) are indicated by letters. Dashed vertical lines separate the evaluated categories. In the graph showing differences within patterns, the dashed lines separate individual acrosomal staining patterns, and statistical comparisons were performed only within each pattern category. In the graph showing differences within sperm groups, the dashed lines separate individual sperm treatment groups, and statistical comparisons were performed only within each group. *n* = 3 independent biological replicates, each replicate consisted of a pooled ejaculate from three boars.

Spermatozoa that successfully undergo IVC and are subsequently exposed to a CaI treatment can complete an in vitro AR, as demonstrated by changes in PNA lectin labeling (Figure 8). Specifically, spermatozoa displaying a ruptured or absent acrosomal PNA signal after CaI treatment (Reacted/No Acr fluorescent signal) are classified as acrosome-reacted. This approach allows for indirect estimation of the proportion of capacitated spermatozoa, as only those that have completed capacitation can respond to CaI by acrosomal exocytosis.

Non-cap, Non-cap + EtOH, Non-cap + CaI, Cap, and Cap + EtOH spermatozoa showed significantly more Whole Acr signal 96.3%, 84.7%, 75.0%, 68.9%, and 70.0%, respectively) than Cap + CaI spermatozoa, in which the No Acr signal predominated in 63.9%. In contrast, the proportion of Reacted Acr spermatozoa remained low in the non-capacitated groups, reaching only 1.6% in Non-cap, 11.2% in Non-cap + EtOH, and 9.5% in Non-cap + CaI. Higher proportions of Reacted Acr spermatozoa were observed after capacitation, accounting for 17.5% in Cap, 17.0% in Cap + EtOH, and 21.7% in Cap + CaI. The Whole Acr pattern still represented the major population in Cap and Cap + EtOH spermatozoa, its proportion was reduced compared with the non-capacitated groups, accompanied by an increase in both Reacted Acr and No Acr patterns. The most pronounced shift was observed in Cap + CaI spermatozoa, in which the Whole Acr signal dropped significantly and the No Acr pattern became the dominant labeling pattern. In our experiments, CaI exposure of in vitro capacitated spermatozoa resulted in a significant increase in the proportion of acrosome-reacted spermatozoa compared with non-capacitated spermatozoa, confirming that capacitation occurred and preceded the induced AR.

### 3.6. Integrated Comparison of Capacitation Markers

To provide an integrated comparison of capacitation-associated markers in boar spermatozoa, we summarize the predominant patterns across methods in Table 1. The table highlights methodological differences and their impact on the interpretation of sperm capacitation status across markers. When all evaluated markers are considered together, a coherent picture of capacitation-associated changes emerges. Although individual techniques differ in sensitivity and biological readout, their combined interpretation supports a coordinated but multi-level remodeling process.

PTyr immunofluorescence revealed a shift from predominantly head-restricted patterns in Non-cap spermatozoa to increased Head + flagellum localization after capacitation, particularly depending on the fixation method. In contrast, Western blot analysis demonstrated a global increase in total PTyr, including a consistent increase in PTyr in the 31 kDa band. These approaches therefore provide complementary information; ICC reflects spatial redistribution of accessible epitopes, whereas Western blot analysis captures overall PTyr intensity at the protein level.

Acrosin immunodetection using ACR.2 antibody showed a transition from predominantly Low signal in Non-cap spermatozoa to a defined Acrosome pattern after capacitation, indicating molecular remodeling within the acrosomal compartment. Importantly, this shift occurred without a parallel increase in spontaneous acrosome loss as assessed by PNA labeling. The majority of spermatozoa retained intact acrosomes after capacitation, and a significant AR was observed only after CaI induction after capacitation. This confirms that capacitation induces biochemical priming for the AR.

Similarly, CTC staining demonstrated a transition from the Whole head pattern (Non-cap) to the Acrosome pattern (Cap), reflecting redistribution of calcium during the capacitation process. The calcium dynamics, protein phosphorylation, and acrosomal protein remodeling occur in parallel but represent distinct layers of the capacitation cascade.

## 4. Discussion

This study was designed to critically evaluate commonly used approaches to detecting sperm capacitation, with a focus on methodological specificity, limitations, and the interpretative boundaries of individual detection techniques. Capacitation is a complex process regulated by both signal stimuli from the surrounding environment and intracellular signals. The pathways leading to capacitation are regulated at multiple levels. These pathways interact and converge to induce structural and functional changes in sperm, thereby preparing them for successful fertilization [18,23,28]. Various detection markers reflect the physiological and biochemical changes occurring in spermatozoa during capacitation. However, as demonstrated in previous studies, individual capacitation markers do not progress synchronously and reflect distinct events; so, it is better to interpret them as complementary rather than independent and unambiguous markers of capacitation [15,29].

Immunocytochemical (ICC) detection of PTyr provides information on the spatial localization of phosphorylated epitopes in individual spermatozoa. Still, it is inherently limited by its strong dependence on fixation and permeabilization conditions [23]. Using aldehyde fixatives or native labeling, ICC primarily detects epitopes accessible on or near the plasma membrane and therefore reflects localized phosphorylation events rather than the overall phosphorylation status of the cell [30,31,32]. In contrast, fixation with alcohol fixatives allows for high permeabilization, making it possible to detect epitopes inside the cell. However, this type of fixation disrupts the membrane; so, the plasma membrane state is not clearly reflected [33,34]. As a result, ICC-based PTyr patterns are highly sensitive to methodological parameters and should be interpreted primarily in spatial and qualitative contexts. The identification of up to nine PTyr fluorescence patterns across fixation conditions in this study further illustrates this methodological variability. While some heterogeneity may reflect genuine biological redistribution of phosphorylated proteins during capacitation, the high pattern diversity is more plausibly explained by differential epitope accessibility, extraction of soluble proteins, and altered membrane permeability [35]. Thus, the observed diversity of fluorescence patterns primarily reflects methodological sensitivity rather than distinct biologically defined capacitation states [23]. For this reason, reduction and standardization of fluorescence pattern categories (in this study, to three groups—No signal, Head, and Head + flagellum) are methodologically necessary. Consolidating patterns into biologically interpretable groups minimizes observer bias, improves reproducibility, and facilitates comparison across experiments and laboratories. Based on our results, fixation with 2% methanol-free formaldehyde with 2% BSA appears to be the most suitable protocol for routine PTyr immunofluorescence in boar spermatozoa. This fixation protocol provided the clearest distinction between non-capacitated and capacitated sperm subpopulations, as most non-capacitated spermatozoa showed PTyr signal mainly in the equatorial subsegment. In contrast, capacitated spermatozoa additionally displayed fluorescence in the flagellum.

Western blot analysis captures global changes in PTyr across the entire sperm population, integrating signals from both membrane-associated and intracellular proteins [35,36]. These two approaches therefore report on distinct biological layers of capacitation-related signaling. Importantly, apparent discrepancies between PTyr patterns observed by ICC and changes detected by Western blot do not represent conflicting results but instead reflect the fundamentally different nature of the information provided by each method. Together, these findings emphasize that ICC and Western blot analyses of PTyr are complementary rather than interchangeable, and that they should not be directly compared without considering their methodological and biological contexts.

Fluorescence-based assessment of capacitation using ACR.2 immunodetection, CTC staining, and PNA labeling with CaI-induced AR provided mutually supportive yet mechanistically distinct information about sperm functional status. Detection of acrosin using the ACR.2 antibody revealed a clear shift in fluorescence patterns after capacitation. In the non-capacitated group, 84% of spermatozoa exhibited the pattern typical of non-capacitated cells, whereas only 10% showed a capacitation-associated pattern. Following capacitation, the proportion of spermatozoa displaying the non-capacitated pattern decreased to 38%, while 58% exhibited the capacitated phenotype. This different labeling confirms previous findings [14,37] that acrosin accessibility or localization changes during capacitation, likely reflecting membrane reorganization and preparatory modifications within the acrosomal region rather than full acrosomal exocytosis [38]. The incomplete conversion (i.e., not 100%) is consistent with the physiological heterogeneity of sperm populations and the asynchronous nature of capacitation [38].

A comparable trend was using the CTC assay, which monitors calcium redistribution [39]. In the non-capacitated group, 81.7% of spermatozoa displayed the non-capacitated fluorescence pattern and 18.3% the capacitated pattern. After capacitation in TALP-based medium, this ratio was reversed, with 79.5% showing the capacitation-associated pattern and only 20.5% retaining the non-capacitated signal. Because CTC primarily reflects intracellular Ca^2+^ dynamics and membrane-associated changes [16,40], these data indicate that calcium redistribution occurs in a majority of spermatozoa under capacitating conditions and parallels the changes detected by ACR.2 labeling.

Functional validation was provided by PNA lectin labeling combined with CaI-induced AR. The predominance of the Whole Acr pattern in untreated non-capacitated spermatozoa confirms that most cells retained intact acrosomes under basal conditions. Similar proportions in the Non-cap + EtOH and Non-cap + CaI groups indicate that CaI alone was insufficient to induce extensive acrosomal exocytosis in non-capacitated boar spermatozoa, supporting the concept that capacitation is required for spermatozoa to acquire responsiveness to AR-inducing stimuli [41,42]. In contrast, capacitated spermatozoa exhibited reduced proportions of Whole Acr cells and increased frequencies of reacted acrosomal patterns. This effect was most pronounced in the Cap + CaI group, in which the No Acr pattern became predominant, indicating extensive AR following ionophore treatment. Similar findings have been reported in boar spermatozoa, in which CaI effectively induces acrosomal exocytosis only after capacitation [39]. This high responsiveness confirms that the majority of spermatozoa acquired functional competence to undergo regulated acrosomal exocytosis, a definitive endpoint of capacitation.

Importantly, while ACR.2 and CTC report structural and signaling-associated changes occurring during capacitation, PNA labeling after CaI provides a functional readout of acrosomal responsiveness. The concordance among these methods strengthens the conclusion that capacitation occurred in a substantial proportion of the sperm population. At the same time, the partial overlap between marker-defined subpopulations underscores that capacitation is a gradual and heterogeneous process, and that different fluorescence-based assays capture distinct biological layers of this complex maturation event.

Together with our systematic review [23], which not only demonstrated the possible effect of fixation protocols on the presentation of specific PTyr fluorescent patterns but also showed how spermatozoa-handling methods and capacitation medium composition shape signaling trajectories, the present work shows that an analytical methodology critically determines how capacitation is detected and interpreted. While the former addressed how experimental conditions influence the biological course of capacitation, the current study clarifies how detection strategies shape its readout. Collectively, these studies highlight that both the experimental design and the analytical framework fundamentally influence conclusions about sperm capacitation and underscore the necessity of an integrative, context-aware interpretation.

## 5. Conclusions

Taken together, our findings do not support the concept of a single “gold standard” marker of capacitation. Instead, they demonstrate that individual assays capture different biological dimensions of this multifaceted process and are variably influenced by methodological context. Calcium redistribution (e.g., CTC or alternative ion-sensitive probes) reflects early membrane and signaling events; PTyr detection (by WB or ICC) reports phosphorylation-dependent signaling, albeit with clear methodological constraints; and acrosomal responsiveness (e.g., PNA labeling after CaI induction) provides functional validation of capacitation competence. Rather than proposing a single standardized marker, our data support a modular approach to capacitation assessment, in which the minimal combination of Ca^2+^ dynamics, phosphorylation status, and acrosomal responsiveness is selected according to the specific research question.

## Figures and Tables

**Table 1:** Overview of capacitation-associated markers in non-capacitated and capacitated boar spermatozoa. The table summarizes the predominant fluorescence or biochemical patterns detected by individual markers in non-capacitated (Non-cap) and capacitated (Cap) spermatozoa. For phosphotyrosine (PTyr) fluorescence, the predominant pattern is shown for each fixation condition (Susp., F mf, F, A, M). Western blot (WB) data reflect global PTyr levels and the intensity of the 31 kDa band (relative optical density, ROD). ACR.2 immunolabeling indicates acrosin-specific patterns (Low signal vs. Acrosome). PNA labeling reflects acrosomal integrity before and after calcium ionophore (CaI) induction. CTC data indicate calcium-dependent redistribution patterns. ↑—increase; ↓—decrease.

Marker	Non-Cap Spermatozoa		Cap Spermatozoa	
	Non-Cap Predominant Pattern	Cap Predominant Pattern	Non-Cap Predominant Pattern	Cap Predominant Pattern
PTyr—fluorescence	**Head**	**Head + flagellum**	**Head**	**Head + flagellum**
Susp.	13%	0%	38%	1%
F. mf	98%	0%	19%	62%
F	96%	0%	11%	63%
A	87%	0%	43%	34%
M	82%	0%	60%	36%
PTyr—WB	**↓ overall PTyr, ↓ PTyr at 31 kDa** 1 ROD, 0.03 ROD	**↑ overall PTyr,** **↑ PTyr at 31 kDa** N/A	**↓ overall PTyr, ↓ PTyr at 31 kDa** N/A	**↑ overall PTyr, ↑ PTyr at 31 kDa** 2 ROD, 0.18 ROD
ACR.2	**Low signal** 84%	**Acr.** 10%	**Low signal** 38%	**Acr.** 58%
PNA	**Whole Acr.** 96.3%	**No Acr. After CaI** 15.5%	**Whole Acr.** 68.9%	**No Acr. After CaI** 63.9%
CTC	**Whole head** 81.7%	**Acr.** 18.3%	**Whole head** 20.5%	**Acr.** 79.5%

## Data Availability

The data that support the findings of this study are available in Section 2 and Section 3 of this article. The data that support the findings of this study are available on request from the corresponding author.

## Abbreviations

The following abbreviations are used in this manuscript:

A	Acetone
Acr	Acrosome
ALH	Amplitude of lateral head displacement
AR	Acrosomally reacted
BSA	Bovine serum albumin
CaI	Calcium ionophore
cAMP	Cyclic adenosine monophosphate
Cap	Capacitated
CASA	Computer-assisted sperm analysis
CatSper	Cation channel of sperm
CM	Capacitation media
CTC	Chlortetracycline
EqSS	Equatorial subsegment
F	Formaldehyde
F mf.	Formaldehyde methanol-free
HVCN1	Voltage-gated hydrogen channel 1
ICC	Immunocytochemistry
IVC	In vitro capacitation
LIN	Linearity
M	Methanol
Non-cap	Non-capacitated
PBS	Phosphate-buffered saline
PKA	Protein kinase A
PNA	Peanut agglutinin
PTyr	Phosphotyrosine
PVA	Polyvinyl alcohol
ROD	Relative optical density
RT	Room temperature
SEM	Standard error of the mean
Susp.	Suspension
TALP	Tyrode’s albumin lactate pyruvate
UV	Ultraviolet
VCL	Curvilinear velocity
